# Synthesis of Glycosides of Glucuronic, Galacturonic and Mannuronic Acids: An Overview

**DOI:** 10.3390/molecules16053933

**Published:** 2011-05-10

**Authors:** Anne Wadouachi, José Kovensky

**Affiliations:** Laboratoire des Glucides UMR CNRS 6219, Université de Picardie Jules Verne, 33 Rue Saint Leu, F-80039 Amiens, France

**Keywords:** glycosidation, glucuronic acid, galacturonic acid, mannuronic acid, stereoselectivity

## Abstract

Uronic acids are carbohydrates present in relevant biologically active compounds. Most of the latter are glycosides or oligosaccharides linked by their anomeric carbon, so their synthesis requires glycoside-bond formation. The activation of this anomeric center remains difficult due to the presence of the electron-withdrawing C-5 carboxylic group. Herein we present an overview of glucuronidation, mannuronidation and galacturonidation reactions, including syntheses of prodrugs, oligosaccharides and stereochemical aspects.

## 1. Introduction

Uronic acids are reducing sugars of biological relevance. They are involved in the metabolism of many drugs and endogenous compounds, and they are found natural products such as glycosaminoglycans, pectins and carragenans, among others, isolated from different sources – mammals, plants and algae. 

In uronidation reactions, uronic acids are attached to an aglycone through the anomeric carbon atom forming *O*-glycosidic bonds. Although sharing all common aspects with general glycosidations, which has been extensively reviewed [1,2], the synthesis of uronic acid glycosides is particularly challenging, because the presence of the C-5 carboxylic group decreases the reactivity at the anomeric position. Different methodologies have been investigated in order to overcome this drawback and to develop general strategies allowing the synthesis of uronic acid glycosides of biological importance and of complex oligosaccharides in a regio- and stereoselective manner. 

Several methods are available for the synthesis of glycosides of glucuronic acids, and these can be placed into two broad categories involving either oxidation of the corresponding glucoside or by carrying out the glycosidation on an activated glucuronic acid. This manuscript covers the second approach.

As glucuronidation has been previously reviewed in 1998 [3,4], the present review will summarize the latest advances on glucuronic, galacturonic and mannuronic acid glycosylation methodologies, especially those involving the synthesis of metabolites of bioactive molecules and oligosaccharides. As L-iduronic acid has been the subject of a great number of articles dealing to the synthesis of heparin sequences, its reactions are out of the scope of this review. 

## 2. Glucuronidation

### 2.1. Metabolites Synthesis

Many drugs have been conjugated to D-glucuronic acid (GlcA) in order to obtain the required tools for improving insights on their absorption, metabolism and bioavailability. Moreover, the isolation of the metabolites is often tedious and analytical standard are necessary as reference compounds for quantification of metabolite levels in clinical samples and for further pharmacological evaluation.

Different methods, for the preparation of these standards conjugated to polyphenol residues, have been developed [1,5,6]. The study of metabolites of drugs can contribute to the toxicity, research and safety assessment, taking into account that the biological activity of GlcA conjugates is often similar or even higher than that of the aglycone (drug) [7,8].

**Scheme 1:**
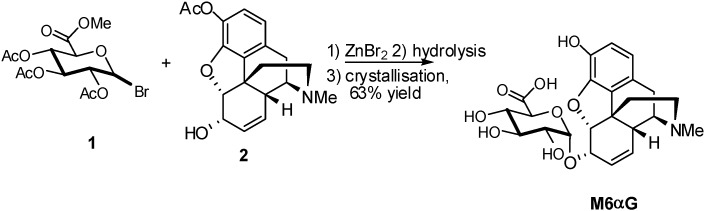
Glucuronidation of 3-*O*-acetylated morphine.

A number of GlcA donors have been used for the synthesis of glucuronides. The first report on stereoselective synthesis and characterization of morphine 6-α-D-glucuronide (M6αG), useful as a reference marker for testing the purity and stability of the pharmaceutically important morphine 6-β-D-glucuronide (M6G) was described by Rukhman *et al*. [9,10] Several groups reported various methodologies for the synthesis of M6G, the preparation of the α-isomer had not been previously reported, and therefore the chemical and biological properties of morphine 6-α-D-glucuronide remained unknown. The synthesis the α-anomer is based on the glycosylation of 3-*O*-acetylated morphine **2** with methyl 2,3,4-tri-*O*-acetyl-D-glucopyranosyluronate bromide **1** as glycosyl donor and zinc bromide as catalyst (Scheme 1).

The selectivity of this reaction is controlled by the amount of catalyst, the use of 1.8 equivalents of ZnBr_2_ afforded M6αG in a 8:1 α/β ratio. After hydrolysis of the acetyl groups, crystallisation gave the α anomer in reasonable yield (63%).

**Scheme 2:**
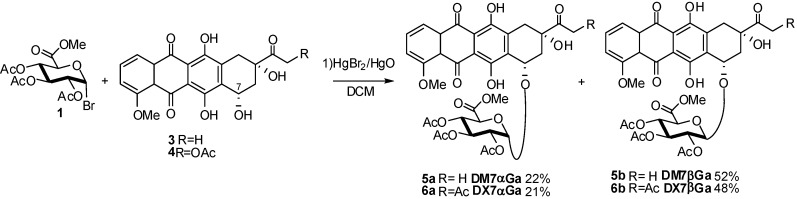
Koenigs-Knorr reaction for synthesis of daunomycin and doxorubicin metabolites.

Other examples of Koenigs-Knorr procedures to synthesize metabolites are the preparation of doxorubicin, daunomycin, clenbuterol and edaravone glucuronates. Daunomycinone-7-D-glucuronide (DM7G, **5**) and doxorubicinone-7-D-glucuronide (DX7G, **6**) were conveniently prepared through the glycosylation at the 7-hydroxyl group of daunomycinone (**3**) or 14-acetoxydoxorubicinone (**4**) with α-glucosyluronate bromide **1** by a Koenigs-Knorr procedure catalyzed by HgBr_2_ (Scheme 2), followed by alkaline deacetylation using aqueous LiOH solution and Amberlite cation exchange material [11]. The desired compounds **5-6** were obtained as a 3:7 α/β mixture, the anomers could be separated by flash column chromatography.

**Scheme 3:**
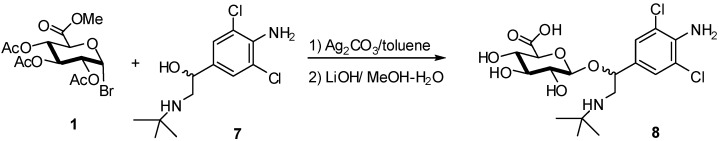
Synthesis of clenbuterol *O*-glucuronide derivatives.

Clenbuterol is a sympathomimetic agent that selectively activates β2-receptors. It is used as a bronchodilator for the treatment of asthma and chronic obstructive pulmonary disease in both human and veterinary medicines. Two clenbuterol *O*-glucuronide diastereomers (**8**, Scheme 3) were synthesized by the Koenigs–Knorr reaction [12] of **1** with racemic **7**. HPLC purification allowed to isolate both diastereoisomers albeit in very low yield (1.7%), due to the formation of orthoesters by-products.

**Scheme 4:**
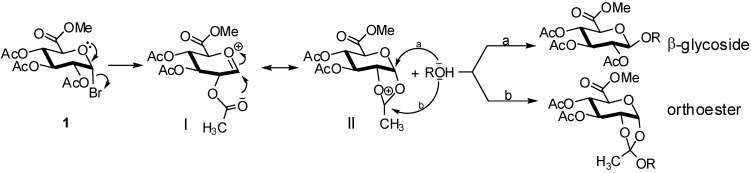
Mechanism of β-glycoside and orthoester formations.

During the the Koenigs Knorr reaction, orthoesters are frequently produced as by-products. The formation of orthoester derivatives can be explained by the competitive nucleophilic attack of the oxygen atom of the alcohol on the two possible electrophilic sites of the intermediate **II**, which is obtained from the oxocarbenium ion **I** (Scheme 4). 

**Scheme 5:**
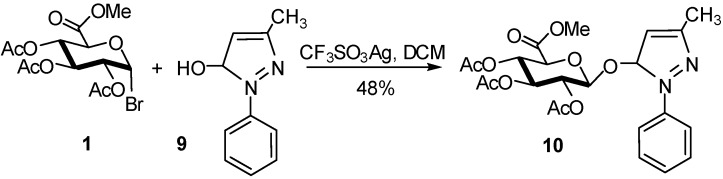
Synthesis of edaravone glucuronate **10**.

Edaravone, a neuroprotective agent is metabolized to the glucuronate metabolite in humans. Edaravone glucuronate and edaravone sulfate, two metabolites of edaravone, were synthesized in high yields [13]. The edaravone glucuronate **10** was synthesized from glucosyluronate bromide **1** by conjugation with edaravone (**9**) using silver trifluoromethanesulfonate as promoter (Scheme 5).

**Scheme 6:**
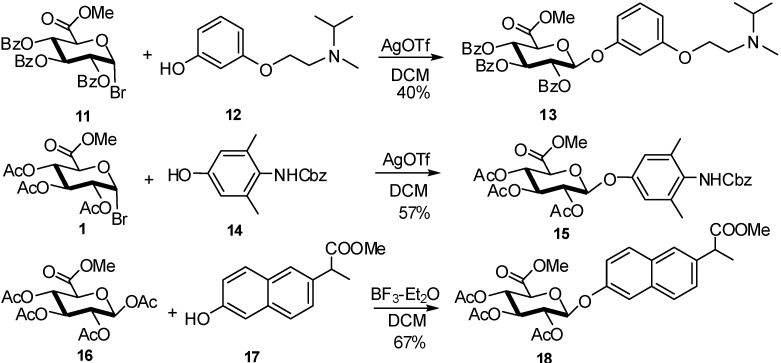
Benzoylated or acetylated glucosyluronate donors.

The investigation of drug metabolism requires a substantial amount of metabolites. As isolation from urine is long and tedious, the material obtained by synthesis is often preferred. In the case of phenolic compounds, the synthesis of glucuronides has been studied for each individual case by Arewang *et al* [14]. A number of GlcA donors as benzoylated or acetylated glucosyluronate bromides were treated with acceptor **12** under silver triflate promotion. Coupling of the acetylated donor **1** with **12** at 0 °C, gave a α/β-mixture (1:3) in 40% yield, whereas the benzoylated donor **11** produced a mixture of β-glycoside **13** and the corresponding orthoester in similar yield at the same temperature (Scheme 6). Exclusive formation of β-glycoside **13**, albeit still only in a moderate yield (40%), was obtained when the coupling between donor **11** and acceptor **12** was carried out at ambient temperature. The use of the other donors did not improve the yield. 

For the aglycone **14**, the acetylated donor **1** gave the best result. On the other hand, in the case of compound **17** the use of 1-*O*-acetyl derivative **16** as donor in the presence of BF_3_ etherate afforded the glucuronide **18** in 67% yield (Scheme 6). These synthetic pathways used easily available glycosyl donors and allowed the preparation of substantial amounts of the target glucuronides. Bromide glucosyluronate donors gave moderate yields and have been compared to imidate glucuronyl donors which are more efficient in most cases. 

**Scheme 7:**
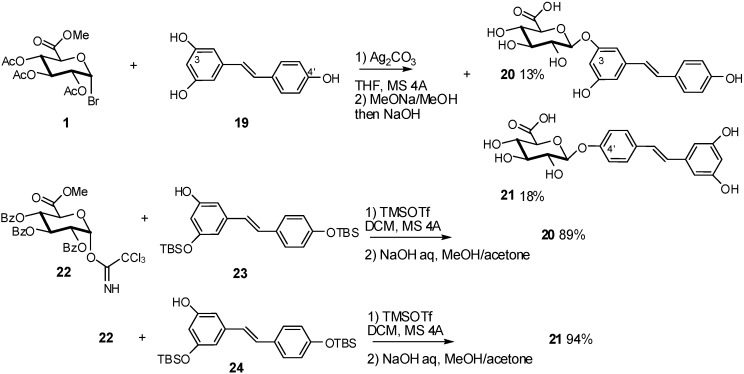
Synthesis of resveratrol-3-*O*- and 4’-*O*-glucuronides **20** and **21.**

To study the resveratrol metabolites and their effects on cell viability and on the inhibition of HIV infection, a one-pot synthetic approach using a random glycosylation procedure between resveratrol (**19**) and methyl acetobromoglucuronate **1** was used leading to resveratrol 3-*O*-glucuronide **20** and resveratrol-4’-*O*-glucuronide **21** [15] (Scheme 7). The 3-*O*- and 4’-*O*-glycoside derivatives were formed in one-pot. After deprotection, the mixture was purified by HPLC and the desired resveratrol-3-*O*- and 4’-*O*-glucuronides **20** and **21** were isolated in 13 and 18% yields, respectively, based on the resveratrol used. 

In order to improve the yields of these two conjugates, the glycosylation between the trichloroacetamidate donor **22** and the silylated resveratrol acceptors **23** and **24** was performed in the presence of trifluoromethanesulfonate. Resveratrol-3-*O*- and 4’-*O*-glucuronides **20** and **21** were obtained in 94 and 89% yields, respectively (Scheme 7).

**Scheme 8:**
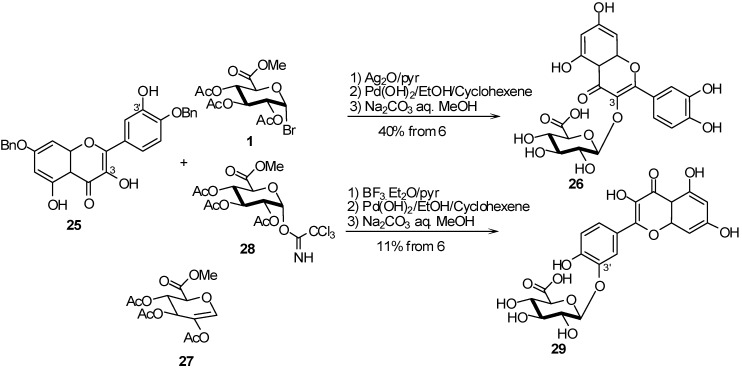
Comparison of bromide and imidate donors for the preparation of quercetin 3’-glucuronides.

Another example is the chemical synthesis of quercetin 3’-glucuronide **29**. Previously described by Wagner *et al.* [16], glucuronidation of **25** with glucosyluronate bromide (**1**) in the presence of silver oxide (Ag_2_O), only gave **26** in 40% yield from **25**. The glucuronidation was accompanied by the formation of the glycal **27** (Scheme 8). The use of imidate donor **28** led exclusively to the quercetin-3’-glucuronide **29** in 11% overall yield. The methyl ester trichloroacetimidate **28** was prepared using a known procedure starting from D-(+)-glucurono-3,6-lactone in 60% yield over four steps [17].

**Scheme 9:**
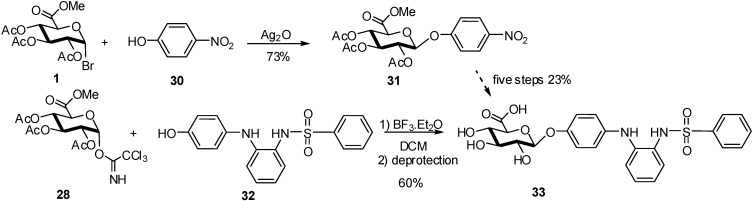
Glucuronidation of ABT-751.

A comparison of the reactivity between bromide and imidate donors was studied for the glucuronidation of ABT-751 **32** which was evaluated as a treatment for pediatric neuroblastoma [18]. Compound **32** is metabolized in humans to glucuronide **33** and therefore the synthesis of both compounds was required to support Phase II clinical studies. The initial synthesis of glucuronide **33** used *p*-nitrophenol **30** and the glucosyluronate bromide (**1**) to form the corresponding glycoside, followed by five further steps to reach the target molecule **33** (Scheme 9).

The Schmidt trichloroacetamidate methodology, promoted by BF_3_.Et_2_O gave, after deprotection, the glucuronide **33** directly in 60% yield, allowing a faster synthesis of the multigram quantities required for clinical use (Scheme 9).

While methods to synthesize simple glucuronides are relatively well developed, the synthesis of structurally complex glucuronides is not straightforward. The efficiency and scale ability of such syntheses is often limited by low yields or unselective glycosidic couplings, complex protecting group strategies, tedious isolations, or enzymatic reactions. In these examples glucuronate imidate donors showed to be more reactive, leading to better results. 

**Scheme 10:**
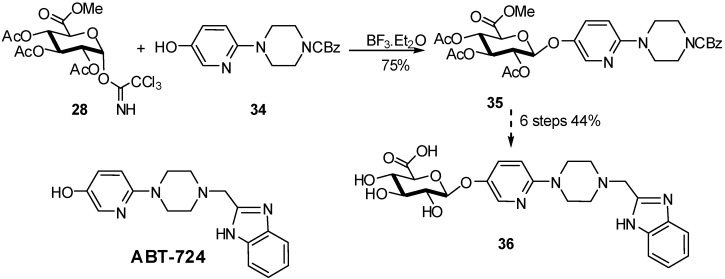
Preparation of the ABT-724 metabolite.

In general, the use of GlcA derived glycosyl donors is often inefficient due to the destabilizing effect of the C-5 electron withdrawing group on the glycosidic bond forming event. A gram-scale synthesis of the glucuronide metabolite of ABT-724, potent selective D_4_ dopamine receptor agonist, could be obtained from imidate donor **28** and compound **34** in the presence of BF_3_.Et_2_O in 75% yield [19]. Compound **35** gave after 6 steps the metabolite of ABT-724 **36** in 33% overall yield from **28** (Scheme 10).

**Scheme 11:**
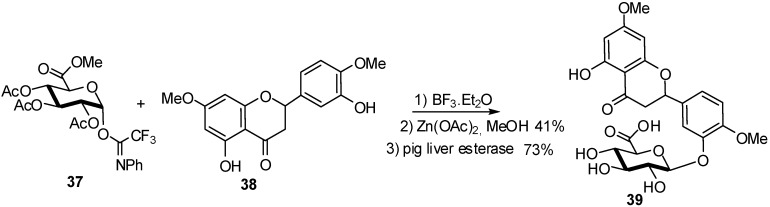
Glucuronidation of persicogenin.

The attempts to synthesize **36** directly from the ABT-724 phenolic compound failed whatever the conditions (donors, promoters) used. For other phenolic compounds, the glucuronidation needs to be compatible with the stability of the aglycone ring as flavones or isoflavones. For example, the first efficient synthesis of flavanone glucuronides as potential human metabolites was optimized for 7,4’-di-*O*-methyleriodyctiol (persicogenin, **38**) because it did not involve a complex protection/deprotection strategy of the aglycone moiety [20]. Thus, the 2,3,4-triacetyl-D-methyl-glucuronate-*N*-phenyl)-2,2,2,-trifluoroacetamidate donor **37** was treated with **38** in the presence of BF_3_ etherate to yield the acetylated glucuronide in 41% which gave after deacetylation and deprotection of the methyl ester by an esterase the final compound **39** in 73% yield (Scheme 11).

**Scheme 12:**
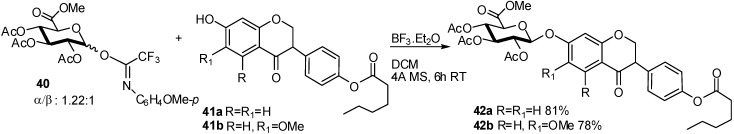
(*N*-*p*-Methoxyphenyl)-trifluoroacetimidate donor for glucuronidation of 4-*O*-hexanoyl-daidzein and glycitein.

A high yielding synthesis of isoflavone 7-glucuronides was accomplished by the reaction between the 7-OH of the isoflavone esters and a novel *O*-acetyl glucuronyl (*N*-*p*-methoxyphenyl)-trifluoroacetimidate donor **40** [21]. Treatment of 4-*O*-hexanoyl-daidzein (**41a**) and glycitein (**41b**) with *O*-acetyl glucuronyl trifluoroacetimidate **40** in CH_2_Cl_2_ under the promotion of BF_3_.Et_2_O (0.2 equiv) at room temperature led to the desired coupling products, **42a** and **42b** in 81% and 78% yields, respectively, as only the β anomers (Scheme 12).

The synthesis of pure standards of isoflavone *O*-glucosides and *O-*glucuronides were developed for a better understanding of the absorption, the metabolism and the bioavailability of isoflavones, [22]. This methodology was used to prepare the 7-*O*-glycosides of the three main isoflavones, daidzein, genistein and glycitein.

**Scheme 13:**
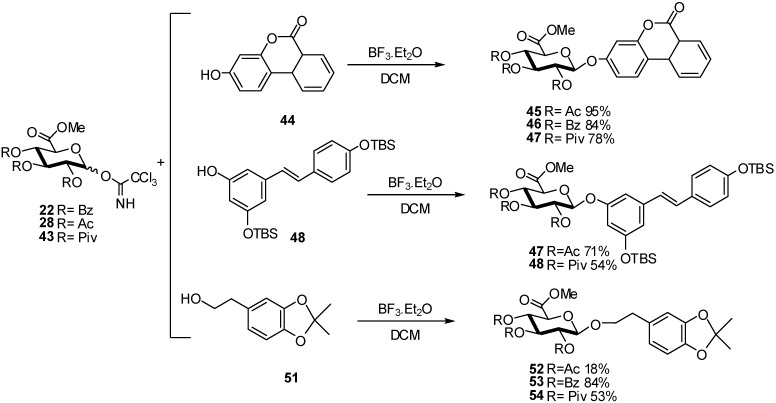
Preparation of isoflavone *O-*glucuronides.

To improve the yields of some glucuronidations of phenolic compounds, different protecting groups on the glucuronate donor were studied. A simple and direct glucuronidation strategy for the urolithin-B **44**, the silylated resveratrol **48**, and the corresponding hydroxytyrosol derivatives **51**, was described (Scheme 13). The critical glycosylation step was optimized using a structurally simple phenol, urolithin-B, by modification of several reaction parameters (solvent, promoter, and glucuronide donor). Glycosylation of urolithin-B acceptor **44** and glucuronosyl donor **43** was first performed using TMSOTf as the promoter with a moderate yield but a very good stereoselectivity, only the β-monomer was obtained. To improve the yield, the most common promoter used in aromatic glycosylation BF_3_.OEt_2_ was used [23] for the reaction of **44** with the glucuronosyl donor **43,** producing compound **47** in much higher yield (78%). When the glucuronosyl donors **22** and **28** were reacted with urolithin-B **44**, products **45** and **46** were obtained in very high yields (95% and 83%, respectively) with no sign of orthoester formation.

The glucuronidation of silylated resveratrol **48** was performed in 71% yield from the acetylated imidate donor **28**. Benzoylated imidate **22** treated with **51** gave a higher yield (84%) than the two other donors **22** and **43** (18 and 53% yield respectively). These results showed the importance of the optimization of the reactions conditions, as well as promoters for each phenolic acceptor/protector donor pair. 

Another protected imidate, the isobutyryl imidate **55,** has been successfully used in this type of reactions. For example, the synthesis of morphine-3,6-di-β-D-glucuronide was efficiently synthesized from this imidate donor. Previous attempts to couple methyl 2,3,4-tri-*O*-acetyl-1-*O*-trichloroacetimidoyl-α-D-glucopyranuronate (**28**) to 3-acetylmorphine by Lewis acid catalysis, afforded mostly 3,6-diacetylmorphine and a small amount of the desired 6-glucuronate. Similar poor results were obtained with morphine **56** [24]. The methyl groups of the sugar acetates was replaced by larger groups in order to increase steric hindrance. Therefore, the rate of nucleophilic attack at the carbonyl, and hence transacylation, was reduced, whereas the rate of glycosylation was relatively unaffected. The isobutyrate group was found to be the best compromise, combining minimal transacylation with ease of hydrolysis. Subsequent use of the tri-isobutyrate **55** led to effective preparations of M3,6diG **57** and related derivatives, with essentially complete stereoselection for the β-anomers due to participation of the neighbouring C-2 acyl group. 

**Scheme 14:**
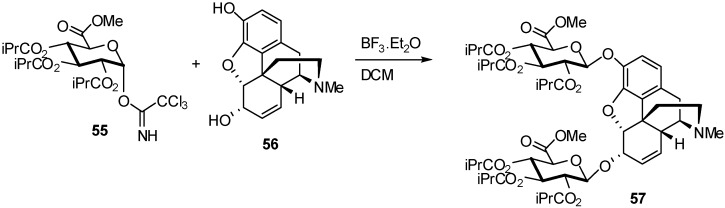
Isobutyryl imidate as donor in the synthesis of morphine-3,6-di-β-D-glucuronide.

By reaction of imidate **55** with dry morphine **56** in dichloromethane in presence of BF_3_/Et_2_O catalyst, the morphine 3,6-β-D-glucuronide derivative **57** in crystalline form was obtained with exclusive β-stereochemistry at C-1 of both glucuronates in 60% yield (Scheme 14).

**Scheme 15:**
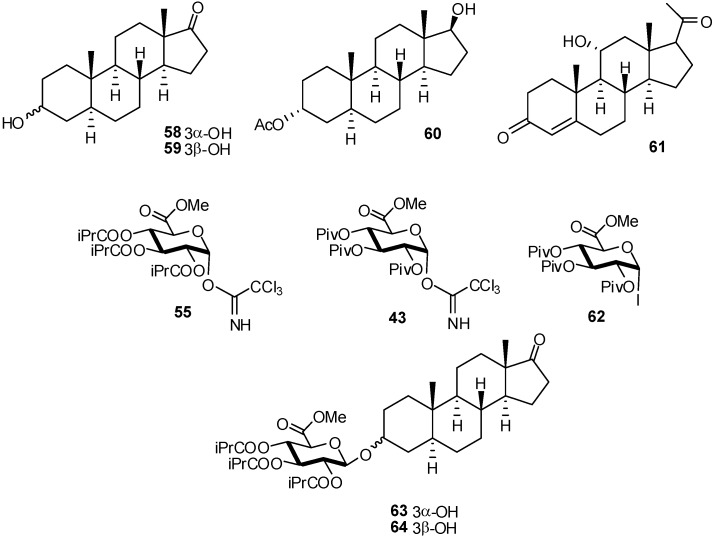
β-Glucuronidation of steroidal secondary alcohols.

The glucuronidation of a number of important steroidal secondary alcohols, such as androsterone (**58**), epiandrosterone (**59**), 17-acetoxyandrostane-3α,17β-diol (**60**) and 11α-hydroxyprogesterone (**61**) was studied for different glycosyl donors [25] (Scheme 15).

Glucuronates are well known to be poor glycosyl donors and the reactivity of alcohols **58**-**61** is rather low. To reduce transacylation, a known side-reaction, reaction of tri-isobutyryl imidate **55** was studied under both ‘normal’ (Method A; *viz.* adding Lewis acid catalyst to the mixture of alcohol and **55**) and ‘inverse’ conditions (Method B; *viz.* adding imidate to a mixture of alcohol and catalyst). The tri-isobutyryl imidate **55** gave satisfactory results in inverse mode with androsterone **58** and epiandrosterone **59,** as compounds **63** and **64** were obtained in 41 and 54% yields respectively, whereas in normal mode the yields were 16 and 34% respectively. Also, imidates **55**, **43** and iodide **62** showed to be efficient donors for the β-glucuronidation of a range of steroidal secondary alcohols.

**Scheme 16:**
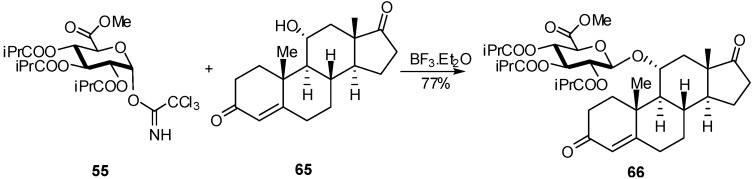
Preparation of estradiol glucuronide.

When acetyl protecting groups were used, orthoesters side-products were obtained, whereas with isobutyryl imidate, in “inverse mode”, the yield increased [26]. For example the glucuronation of estradiol derivative **65** with the imidate **55** gave the glucuronide **66** in 77% yield (Scheme 16).

**Scheme 17:**
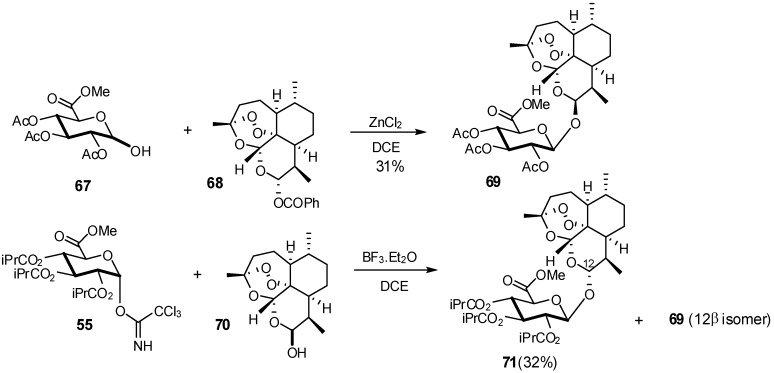
Conjugation of artemisinin and dihydroartemisinin to GlcA.

Studies on the rapid metabolism of the trioxane derivative artemisinin, dihydroartemisinin (DHA, **70**) required its conjugation to GlcA [27]: 

Glucuronidation of **67** as acceptor component may work well if the donor can generate a highly stabilized carbonium ion, though at low temperature the 12α-1’β-glucuronide may predominate. So, when **67** and **68** were treated Use of TMS triflate-AgClO_4_ at −10 °C, the 12α-isomer was obtained in 40% yield as the only product, while the use of BF_3_/Et_2_O at 20 °C gave mainly an anhydro-DHA. However, ZnCl_2_ proved to be an effective catalyst. Thus reaction of **67** with **68** in the presence of ZnCl_2_ afforded crystalline **69** in very satisfactory yield after chromatography (31%) (Scheme 17). The tri-*O*-isobutyryl imidate **55** showed improved stability and reduced transacylation compared to its acetyl protected analog. Thus reaction of **55** and **70** with BF_3_.Et_2_O gave complete reaction of **70** with noticeably less amounts of the DHA degradation products. By chromatography, the 12α,1’β-glucuronide ester **71** was isolated in excellent purity and 32% yield and 15% of the 12β-isomer **69**. Both new esters **69** and **71** gave microanalytically pure material on recrystallization (Scheme 17).

**Scheme 18:**
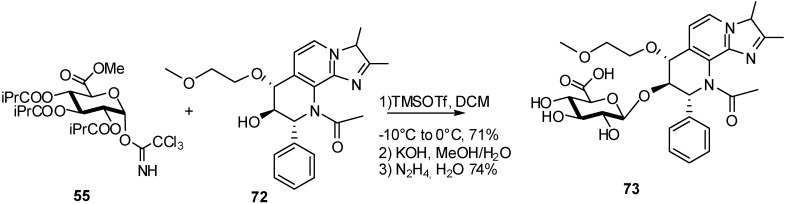
Synthesis of soraprazan glucuronide.

A successful synthesis of the glucuronide metabolite **73** was performed using a *N*-acetylated Soraprazan **72** and tri-iso-butyrate trichloroacetamidate donor **55** [28] (Scheme 18), avoiding the formation of orthoesters observed when using the analogous acetyl protected trichloroacetimidate donor [26].

**Scheme 19:**
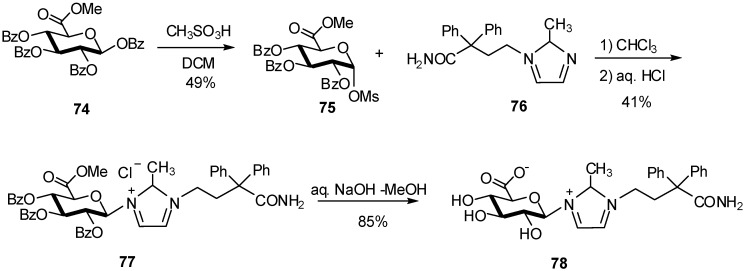
Methanesulfonyl-α-D-glucopyranuronate donor.

Activation of the anomeric position of the glucuronate donor can also be achieved with a sulfonyl group. *N*-glucuronide **78,** a major metabolite of 4-(imidazole-1-yl)butanamide derivative KRP-197/ONO-8025, known for its antimuscarinic activity, was synthesized *via* glucuronidation of compound **76** using methyl 2,3,4-tri-*O-*benzoyl-1-methanesulfonyl-α-D-glucopyranuronate (**75**) [29] (Scheme 19). The latter showed β-selectivity and the glucuronide **77** was obtained in moderate yield (41%). Although this work involved the synthesis of *N*-glycosides, the strategy has a potential application in the preparation of *O*-glycosides from nucleophilic hydroxyl containing acceptors.

This methodology, with formation of an anomeric mesylate, could be applicable to *O*-nucleophiles.

### 2.2. Prodrug Therapy

Since most of the glucuronides exhibit a weaker biological activity than their corresponding aglycones, the glucuronidation is generally considered as an important detoxification metabolic process in mammals. However, even if the glucuronide has no activity itself, it can undergo an enzymatic hydrolysis catalyzed by β-D-glucuronidase, releasing the corresponding biologically active aglycone. In some cases, the glucuronidation can maintain or even increase the therapeutic effect of the drug [30], probably because the active compound is gradually liberated. Synthesis of prodrugs, via a glucuronidation reaction has been studied for the developpement of more selective drugs, specially for selective delivery of systemically administrated chemotherapeutic drugs for solid cancers. 

**Scheme 20:**
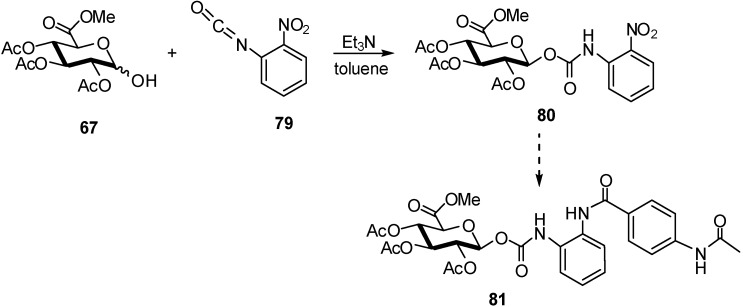
Preparation of β-*O*-glucuronyl carbamate in synthesis of prodrugs.

Indeed, glucuronides can be selectively activated at the tumoural site since the enzyme β-D-glucuronidase is found at highly elevated concentrations in necrotic tumour tissue [31,32]. The design of a suitable glucuronide prodrug must be based upon four criteria: enhanced water solubility, stability in blood, decreased cytotoxicity and drug release after enzymatic cleavage. Several glucuronide prodrugs have already been synthesized and proved to be selectively activated by β-glucuronidase, either present in high concentration in necrotic tumour areas (PMT) [31] or previously targeted to the tumour sites (ADEPT [33], GDEPT [34]), and consequently demonstrated superior efficacy *in vivo* compared to standard chemotherapy [35]. For example, two glucuronide prodrugs of the histone deacetylase inhibitor CI-994 **81** were synthesized [36]. The β-*O*-glucuronyl carbamate **80** was synthesized by coupling the methyl glucuronate **67** with commercially available 2-nitrophenyl isocyanate in a very high β-diastereoselectivity in 86% yield (e.d. 97%) using the method developed by Leenders *et al*. [37] (Scheme 20).

**Scheme 21:**
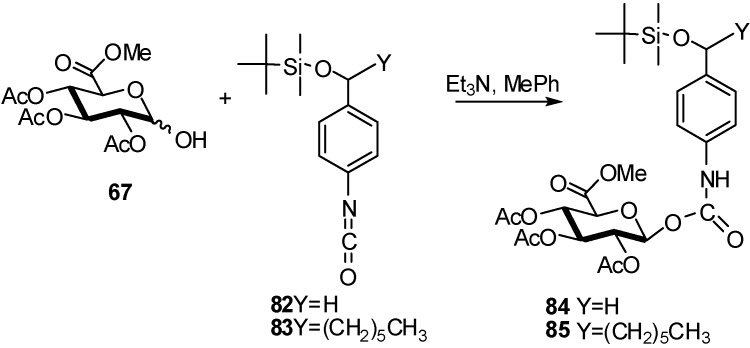
β-Diastereoselective reaction of glucuronyl donor **67** with isocyanate compound.

A series of anthracycline prodrugs containing an immolative spacer were synthesized for application in selective chemotherapy. The key step in the synthesis of all prodrugs is the highly β-diastereoselective addition reaction of the anomeric hydroxyl of a glycosyl donor **67** to a spacer isocyanate resulting in the respective β-glycosyl carbamate pro-moieties [38] (Scheme 21).

**Scheme 22:**
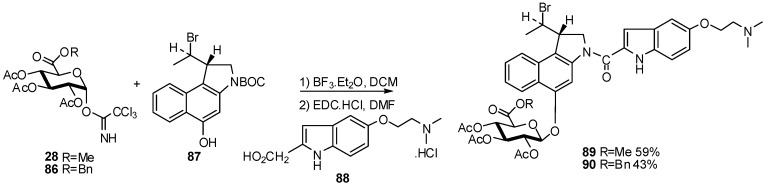
Preparation of duocarmycin glucuronide.

The synthesis and biological evaluation of novel prodrugs based on the cytotoxic antibiotic duocarmycin was realized from imidate donors **28** and **86**. The resulting glucuronide compounds were not isolated and directly coupled with the indole carboxylic acid **88** to afford the corresponding β-glucuronide **89** and **90** in 59% and 43% yields respectively [39] (Scheme 22).

### 2.3. Antibacterials Inhibitors

**Scheme 23:**
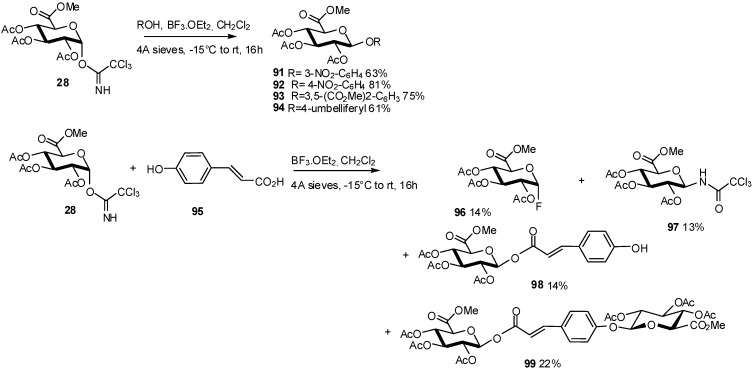
Trichloroacetimidate-activated GlcA as donor in the synthesis of aryl glucuronides.

In the development of aryl glucuronides as potential probes for heparanase, the acid-catalysed glycosidation between the trichloroacetimidate-activated GlcA **28** and a variety of phenols was investigated. In preliminary studies, the BF_3_/Et_2_O-catalysed coupling of imidate **28** with phenols provided the desired aryl glucuronides **91**-**94** in high yields (61–81%) (Scheme 23). The attempted BF_3_.Et_2_O-catalysed glycosidation of **28** with 4-hydroxycinnamic acid **95** did not give the desired glycoside, but instead gave a complex mixture of products **96-99** [40] (Scheme 23).

**Scheme 24:**
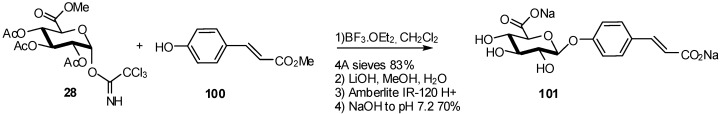
*O*-glucuronidation of methyl 4-hydroxycinnamate.

To obtain the *O*-aryl glucuronide **101** in satisfactory yield, the methyl ester **100** was used as glycosyl acceptor (Scheme 24).

**Scheme 25:**
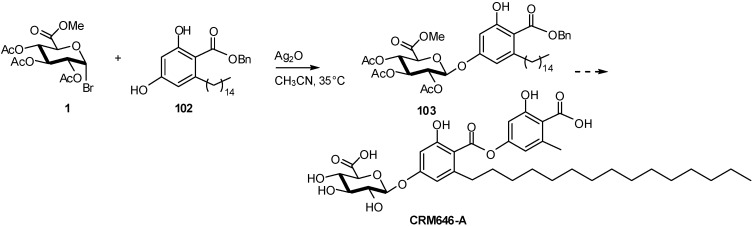
Preparation of two fungal glucuronides.

CRM646-A and -B , two fungal glucuronides with a dimeric 2,4-dihydroxy-6-alkylbenzoic acid (orcinol *p*-depside) aglycone showing significant heparinase and telomerase inhibition activities, were synthesized for the first time [41]. The successful approach involved the construction of the phenol glucuronidic linkage, via coupling of the orsellinate derivative **102** with glucosyluronate bromide **1**, before assembly of the phenolic ester onto the depside aglycone (Scheme 25). Attempts to perform direct glycosylation of the depside aglycone derivatives were not successful.

**Scheme 26:**
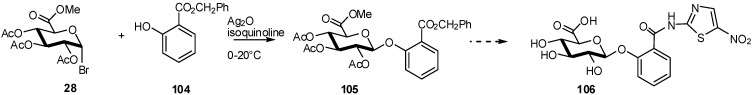
Synthesis of tizoxanide metabolite.

Tizoxanide is a potent antibacterial and antiparasitic agent. Metabolism of tizoxanide leads to the *O*-aryl glucuronide **106** which was efficiently synthesised in four steps from benzyl salicylate **104** and showed a low antibacterial activity. Koenigs-Knorr reaction of **1** with **104** gave the conjugate **105** in 61% yield which was then converted into **106** [42] (Scheme 26).

### 2.4. Methodologies and synthesis of oligosaccharides

To develop the synthesis of biologically active lactone *O*-glucuronides two strategies from 2-*O*-acyl glucuronate donors and glycosylation with more reactive benzyl protected glucuronates were attempted. The anchimeric assistance of an acyl substituent on *O*-2 leading preferably to the β-configuration, whereas stereochemical control of the glycosylation by using ether protected intermediates remained complicated. 

**Scheme 27:**
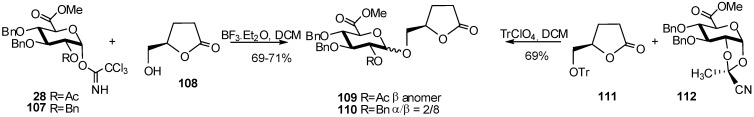
2-*O*-Acyl glucuronate or cyanoester donors for β-glucuronidation.

β-Glucuronated lactone **109** was provided from 2-*O*-acetylated donors **28** or cyano-orthoester **112.** The anchimeric participation of the acetyl group in **28** guarantees the formation of the β-linkage, whereas the tribenzylated donor **107** gave the glucuronide **110** as a 2:8 anomeric mixture [43] (Scheme 27). 

**Scheme 28:**
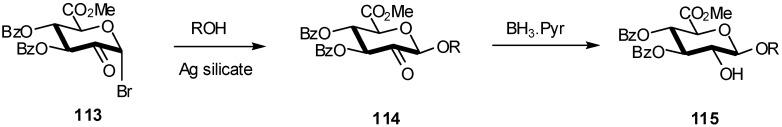
Ulosyl bromide donor for β-glucuronidation.

An indirect strategy for the synthesis of glycosides GlcA involved the use of ulosyl bromide **113** (easily obtained in four steps from glucuronolactone). Glycosylation in the presence of insoluble silver catalyst led to the β-glycoside **114** which could be converted stereoselectively to the *gluco* isomer **115**, whereas selectride reduction afforded the mannuronide derivative [44] (Scheme 28). 

**Scheme 29:**
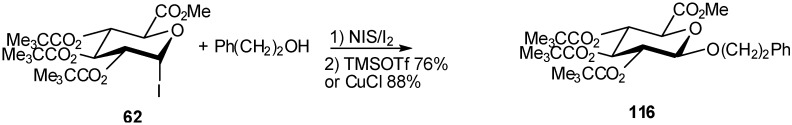
“Disarmed” glucuronyl iodide for β-glucuronidation.

Glucuronyl iodide **62** has been studied as a “disarmed” glycosyl donor and primary or secondary alcohols as acceptors, promotion with NIS/I_2_ followed by TMSOTf gave the corresponding β-glucuronides in good yields [45] (Scheme 29). For example, 2-phenylethanol was glucuronylated in 88% yield when CuCl was used.

**Scheme 30:**
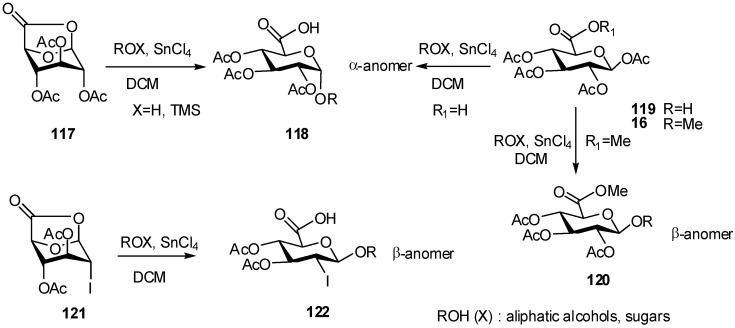
Stereoselective SnCl_4_-catalyzed glucuronidation.

This methodology was applied to the synthesis of disaccharides with the same β-stereoselectivity.Glycosylation of conformationnally inverted donors derived from GlcA was studied towards several silyl ethers [46]. 6,1−Lactone derivative **117** has been used in the synthesis of 1,2-*cis*-glycoside **118**, the SnCl_4_-catalyzed coupling of silyl ethers with **117** provides α-*O*-glucuronides in significantly improved yields without loss of stereoselectivity (Scheme 30). This methodology was extended to 2-deoxylactones, which gave α or β-glycosides depending on the structure of the donor.

The stereoselectivity observed for both **117** and **119** contrasted with that of the methyl ester **16**, which only gave β-glucuronides **120**. Similarly, the 2-deoxy-2 iodo donor **121** gave the β-glycosides **122**, which is explained by the participation of iodine, better than the 2-*O*-acetyl group (Scheme 30).

Cyclic imidates can be used as glycosyl donors, and it was observed that 1,2-*cis* glycosides obtained from the reactions of glycosyl acetates or cyclic imidates, resulted from the anomerisation of initially formed 1,2-*trans* glycosides [47].

**Scheme 31:**
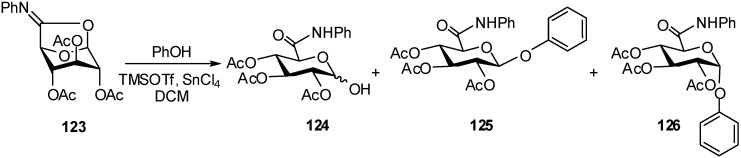
Cyclic imidates as glycosyl donors.

For example, reaction of imidate **123** with phenol in the presence of TMSOTf-SnCl_4_ (2.5/.5) gave after 1h a mixture of compounds **124**, **125** and **126** in 1:1:2.2 ratio, and after 24h only the α-anomer in 63% yield (Scheme 31). The rate of the anomerisation reaction showed to be dependent on the structure of the aglycone [48] and for glucopyranuronic acid the anomerisation is faster than that of glucopyranuronate compounds.

**Scheme 32:**
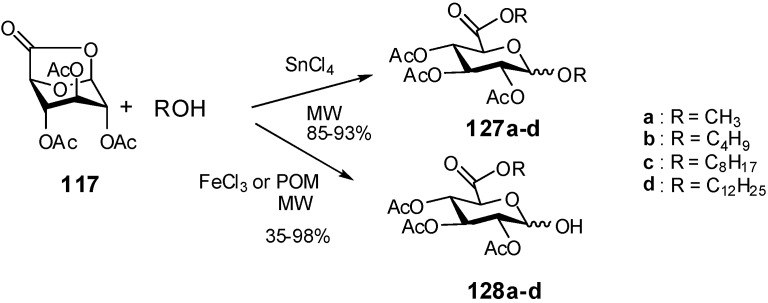
Microwave-assisted glucuronidation.

Microwave-assisted reaction of 6,1-lactone **117** with alcohols in the presence of acidic catalysts has been recently described. Classical Lewis acidic catalysts used in solvent-free conditions under MW irradiation gave different chemoselectivities compared to classical reactions, in shorter reactions times. For example, SnCl_4_ provided glycosylated-esterified compounds **127,** while with FeCl_3_ or [α_1_-M(H_2_O)_4_P_2_W_17_O_61_]^n–^ (M = Yb, Hf and Zr) Dawson-type polyoxometalate (POM) the chemoselectivity was in favour of esterified products **128** [49,50] (Scheme 32). 

**Scheme 33:**
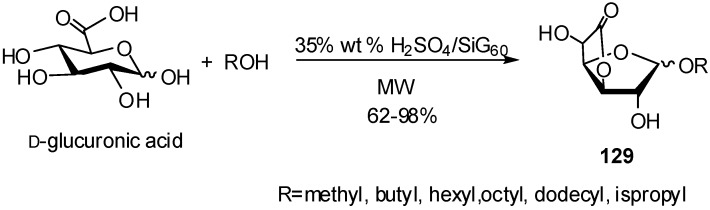
Microwave-assisted formation of alkyl glucofuranosidurono-6,3-lactones.

Other heterogeneous systems were used, sulfuric acid loaded on porous silica (H_2_SO_4_/SiG_60_) and silica-supported Keggin type heteropolyacid. The reaction of the GlcA with different alcohols in the presence of these catalysts, gave glucofuranosidurono-6,3-lactone glycosides **129** in 62–98% yields (Scheme 33). 

The supported sulfuric catalyst was stable under microwave conditions and could be recovered and reused [51]. The formation of alkyl glucofuranosidurono-6,3-lactones have been already described from unprotected GlcA in heterogeneous media and promoted by Lewis acids [52].

The synthesis of the selectively protected disaccharides glycosides **132** and **133**, which are required for further conversion into glycosyl donors for block synthesis of more extended oligosaccharides was studied.

**Scheme 34:**
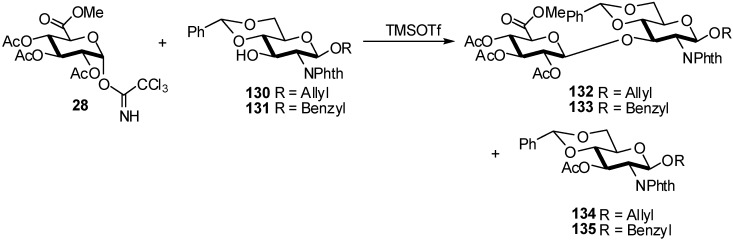
Stereoselective synthesis of disaccharides glycosides.

A strategy for the synthesis of the target disaccharides [53], was the selection of the glucosyluronic donor. Selective *O*-deacetylation of methyl 1,2,3,4-tetra-*O*-acetyl-β-D-glycopyranuronate using hydrazinium acetate gave methyl 2,3,4-tri-*O*-acetyl-D-glucopyranuronate **67**, followed by treatment with trichloroacetonitrile and 1,8-diazabicyclo[5,4,0]-undec-7-ene (DBU) to form the crystalline imidate **28** in good yield. The coupling of compound **28** with **130**, in the presence of trimethylsilyl triflate as the glycosyl promoter and molecular sieves 4 A° in dichloromethane for 2 h at −30 °C, gave the desired disaccharide glycoside **132** in moderate yield (54%), after separation from some accompanying transesterification product of the acceptor, namely allyl 3-*O*-acetyl-4,6-*O*-benzylidene-2-deoxy-2-phthalimido-β-D-glucopyranoside **134** (Scheme 34). Coupling of compound **28** with **131** gave the crystalline glycoside **133** in 59% after separation of the side-product **135**.

**Scheme 35:**
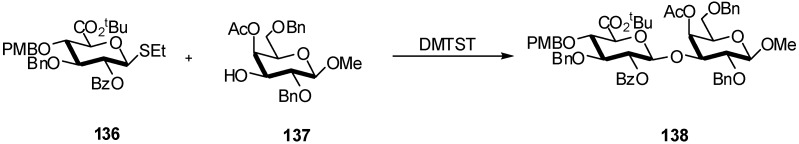
Thioethyl glucuronyl donor.

Westman *et al*. prepared the thioethyl donor **136** for glycosylation of methyl 4-*O*-acetyl-2,6-di-*O*-benzyl-3-D-galactopyranoside (**137**) using dimethyl(methylthio)sulfonium triflate (DMTST) as promoter, affording the disaccharide **138** in 87% yield [54] (Scheme 35). Interestingly, this reaction was performed in the absence of an acid acceptor in order to prevent orthoester formation. 

**Scheme 36:**
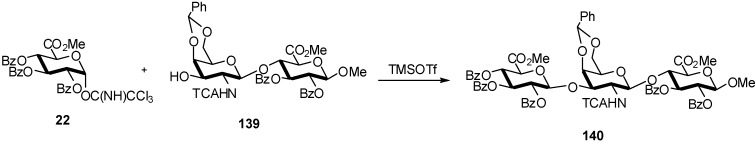
Synthesis of a trisaccharide containing glucuronyl units.

Jacquinet’s group reported that *O*-benzoylated derivatives of GlcA activated through their corresponding trichloroacetimidates were very efficient donors for the preparation of β-D-glucuronides [55]. Condensation of methyl 2,3,4-tri-*O*-benzoyl-1-*O-*trichloroacetimidoyl-α-D-glucopyranuronate **22** (1.5 equivalents) with the alcohol **139** in the presence of trimethylsilyl triflate afforded the crystalline trisaccharide derivative **140** in 85% yield (Scheme 36).

**Scheme 37:**
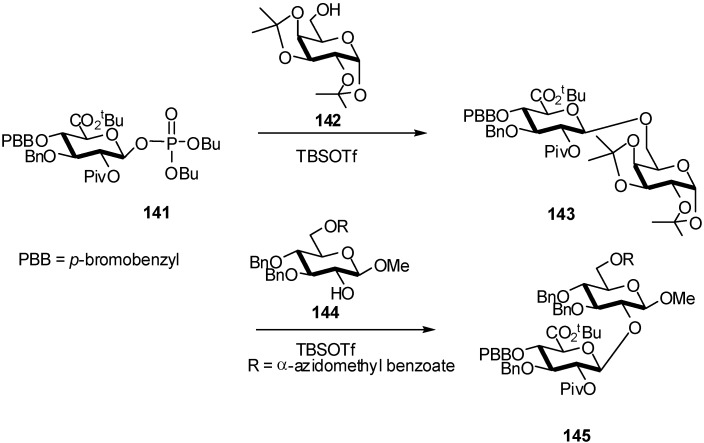
Glucuronidation of alcohols with GlcA phosphate donor.

Deactivated donors such as GlcA phosphate **141** were found to be highly efficient in reactions with primary or secondary alcohols. Combined with the straightforward synthesis from readily accessible GlcA glycal precursors, the use of **141** as a glycosylating agent provided a direct entry to complex glycan structures [56]. The promoters used are TMS or TBS triflates, and the reactions were performed at low temperatures (−50 °C to −20 °C) affording **143** and **145** in 72% and 84% yield, respectively (Scheme 37).

**Scheme 38:**
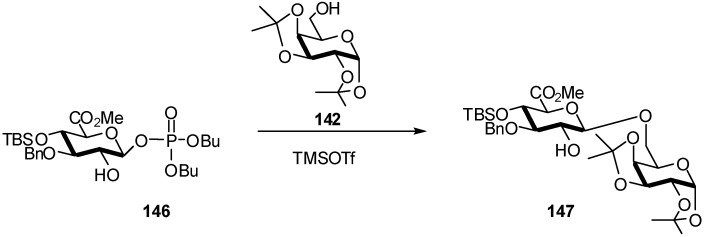
Glycosylation with C2-OH GlcA phosphate donor.

Interestingly, glycosylations performed with C2-OH donor **146** (Scheme 38) also proceeded with complete β-selectivity. GlcA phosphate **146** was prepared and coupled with **142**. The reaction was significantly more rapid than in the case of donor **141** bearing a C2-ester. Disaccharide **147** was obtained as the only product in good yield (65%) (Scheme 38). 

**Scheme 39:**
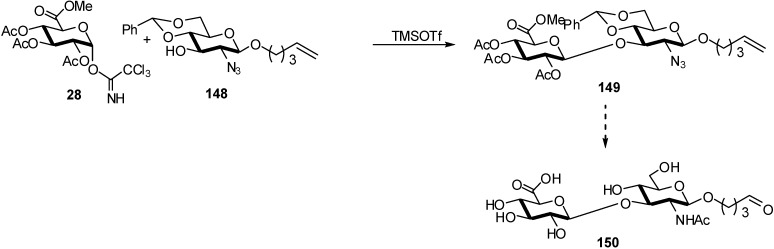
Preparation of synthetic mimics of hyaluronan.

During the preparation of synthetic mimics of hyaluronan and its dimerized (Gemini) disaccharides [57] that could act as versatile building blocks with different therapeutic applications, glycosylation of the *n*-pentenyl glycoside acceptor **148** was carried out with the trichloroacetimidate donor **28** in the presence of TMSOTf , affording the β-(1→3) linked disaccharide **149** in 78% yield (Scheme 39).

**Scheme 40:**
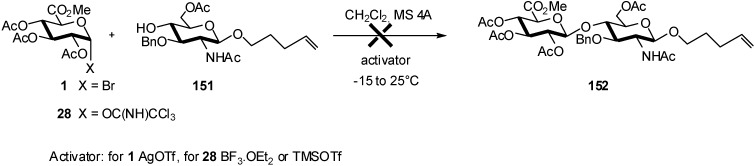
Unreactive *N*-acetylglucosamine acceptors in glucuronidation reaction.

Among others, Rele *et al*. showed that *N*-acetylglucosamine derivatives are unreactive acceptors [58]. Glycosylation of the *n*-pentenyl-terminated *N*-acetyl-D-glucosamine acceptor **151** with either glycosyl donor **1** or **28** was unsuccessful (Scheme 40). 

**Scheme 41:**
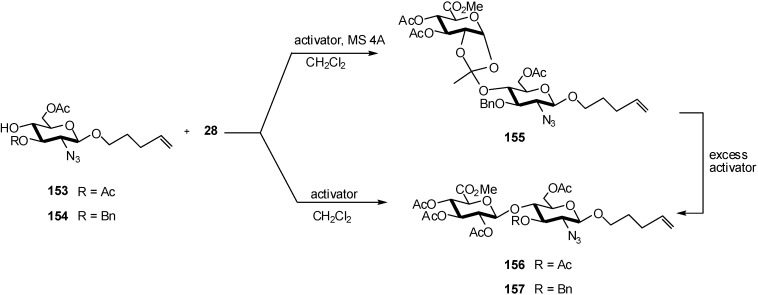
Glucuronidation of *n*-pentenyl glycosides.

On the other hand, azido precursors as **153** or **154** were suitable acceptors. Glycosylation of the *n*-pentenyl glycoside acceptors was carried out separately using the trichloroacetimidate donor **28** in the presence of acid promoters (Scheme 41). Attempts to glycosylate the sugar alcohol **154** with imidate **28** in the presence of TBSOTf or TMSOTf and molecular sieves produced the undesired ortho ester intermediate **155** as the major product. The authors believe that the molecular sieves, which are alumino silicates and are inherently basic in nature, hindered acid-catalyzed 1,2-trans glycosidic bond formation. Indeed, in the absence of molecular sieves, boron trifluoride etherate mediated glycosylation of **153** with imidate **28** afforded the thermodynamically favored *β*-(1,4) disaccharide without a trace of ortho ester, albeit in 30% yield after 14 h. Significantly, TMSOTf-catalyzed conditions provided the most efficient route for coupling the acceptor **154** with glycosyl donor **28**, producing the fully protected disaccharide **157** in moderate (60%) yield. The ortho ester **155** could be converted to disaccharide **157** using an excess amount of acid catalyst in the absence of molecular sieves. It was also observed that replacing the 3-*O-*benzyl protecting group by a 3-*O-*acetyl functionality retarded *β*-(1,4) glycosidic bond formation. Likely, the electron-donating nature of the 3-*O-*benzyl group enhanced the nucleophilicity of the glycosyl acceptor at the 4 position.

**Scheme 42:**
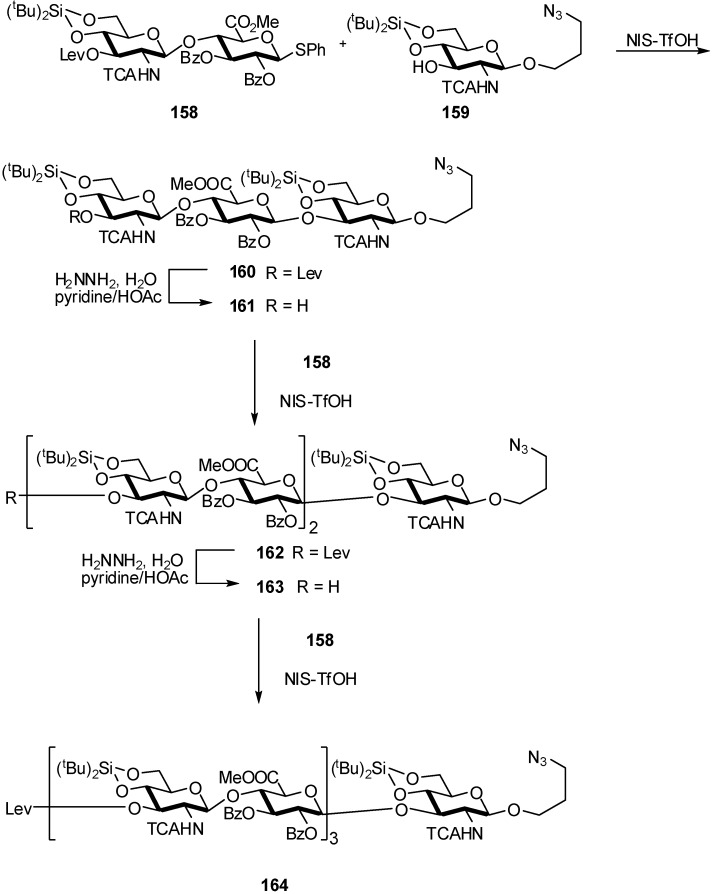
Synthesis of hyaluronic acid-oligosaccharides.

Thiophenyl glucuronate disaccharide donnor **158** was used by Dinkelaar *et al*. [59] in an iterative strategy for hyaluronic acid (HA)-oligosaccharides assembling (Scheme 42). First, the reducing end glucosamine **159** was condensed with dimer **158** using the Ph_2_SO/Tf_2_O activating system. Although preactivation of the thiodisaccharide proceeded smoothly, the ensuing reaction with acceptor **159** did not go to completion and trisaccharide **160** was isolated in 46% yield. Changing from Ph_2_SO/Tf_2_O to the related BSP/Tf_2_O reagent system significantly improved the outcome of the glycosylation, allowing to obtain the protected hyaluronic acid trisaccharide **160** in 75% yield. *N*-iodosuccinimide (NIS)/TfOH as activator system was also examined, giving trisaccharide **160** in 75% yield. Interestingly, the NMR spectrum of **160** revealed a rather small homonuclear coupling constant (*J*H1′-H2′) for the anomeric proton of the glucuronate moiety (H-1′) of 4.4 Hz. Upon deprotection of the oligosaccharides the coupling constant changed to 8.4 Hz, indicative of the β-glucuronic acid linkage formed. The small coupling constant for the glucuronate anomeric proton suggested that the glucuronate ester takes up a flattened 4C1-chair conformation, when positioned in between two 4,6-*O*-di-*tert*-butylsilylidene glucosamine residues. To elongate trisaccharide **160**, the C3′′-*O*-Lev was deprotected and the resulting alcohol **161** was condensed with dimer **158** (NIS/TfOH activation) and pentamer **162** was obtained in 98% yield. Ensuing delevulinoylation of **162** gave alcohol **163** which was elongated in a subsequent NIS/TfOH mediated glycosylation with building block **158**. Heptamer **165** was easily separated from the smaller products in the reaction mixture by size-exclusion chromatography on Sephadex LH-20, and isolated in 61% yield.

**Scheme 43:**
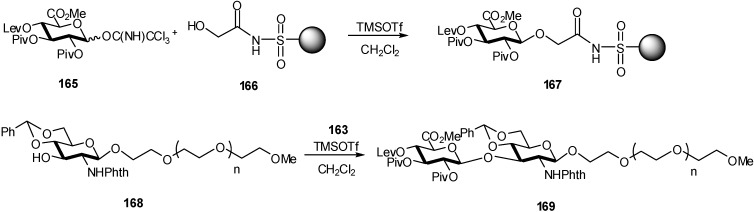
Supported synthesis of HA oligosaccharides.

In a investigation of the use of a safety-catch linker for supported synthesis of HA oligosaccharides, de Paz *et al*. [60] performed the glycosylation of acceptor **166** with donor **165** affording polymer-bound GlcA derivative **167** (Scheme 43). The reaction was repeated to drive it to completion as the first cycle resulted only in partial glycosylation.

Unfortunately, after delevulinoylation, the corresponding alcohol failed as acceptor with a glucosamine donor for disaccharide synthesis. The same drawback was encountered when a glucosamine acceptor was fixed on the resin and glycosylation with **165** was tried. This problem was attributed to the acylsulfonamide linker, and model glycosylations were carried out in solution and on PEG support without the *N*-acylsulfonamide linker to demonstrate this hypothesis. Thus, polymer acceptor **168** was efficiently glycosylated with trichloroacetimidate **165** to afford bound disaccharide **169**. Therefore, the safety-catch linker approach was not suitable for oligosaccharide assembly involving glycosylation of low nucleophilic acceptors with electron-poor donors. It is reasonable to suppose that the chemical nature of the linker, in particular the high acidity of the NH proton, can explain the presence of charged species that hinder coupling reactions mediated by oxocarbenium ions. 

**Scheme 44:**
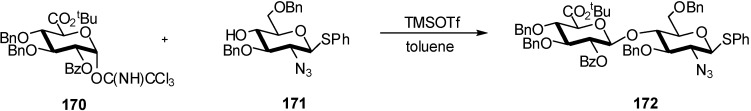
Preparation of a key disaccharide building block for synthesis of oligosaccharides.

The synthesis of glycosaminoglycan oligosaccharides has been the main interest of several research groups. Concerning heparan sulfate oligosaccharides, the synthesis of the key disaccharide building block **172** was smoothly accomplished by TMSOTf-catalyzed reaction (−30 °C) of the 2-*O*-benzoyl-protected GlcA imidate **170** with the azido acceptor **171**, providing the desired disaccharide donor **172** in 89% yield [61] (Scheme 44). 

This block was used for the synthesis of a tetrasaccharide involved in prion diseases. The enzymatic synthesis of GlcA glycosides by the use of snail (*Helix pomatia* and *Helix aspersa*), limpet (*Patella vulgata*), and bovine glucuronidases was investigated by Nagatsukaa *et al.* [62]. 

**Scheme 45:**
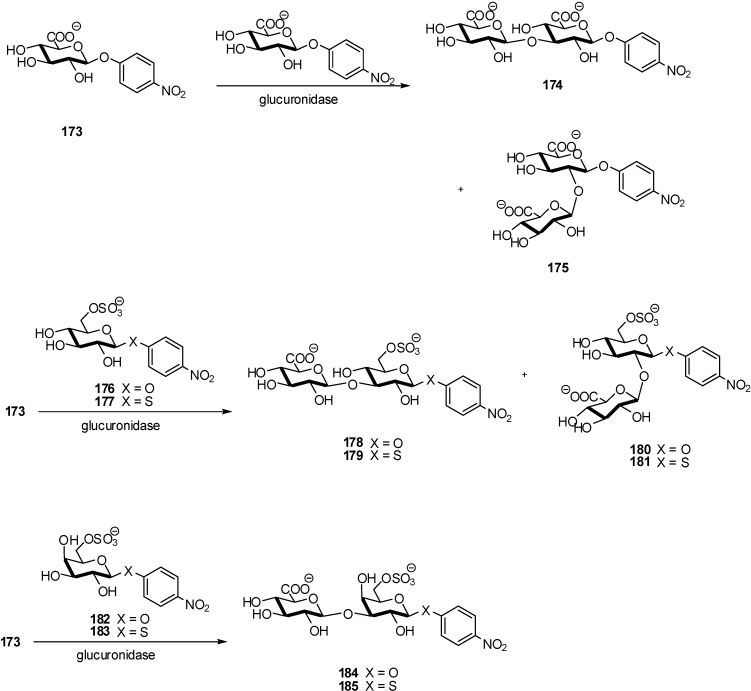
Enzymatic synthesis of oligosaccharides.

As the acceptors, GlcA-*O*-*p*NP **173** (for selftransglycosylation), 6-*O*-sulfo-*β*-V-glucopyranosides (6-*O*-sulfo-Glc-*O*-*p*NP **176** and 6-*O*-sulfo-Glc-*S*-*p*NP **177**), and 6-*O*-sulfo-*β*-D-galactopyranosides (6-*O*-sulfo-Gal-*Op*NP **182** and 6-*O*-sulfo-Gal-*S*-*p*NP **183**) were employed, using GlcA*O*-*p*NP as the donor substrate (Scheme 45). All of the snail, limpet, and bovine enzymes were able to transfer GlcA from GlcA-*O*-*p*NP to the *O*-2 and *O*-3 positions of 6-*O*-sulfo-*β*-D-glucopyranoside.

The limpet and bovine enzymes can also use the *O*-3 position of 6-*O*-sulfo-*β*-D-galactopyranosides for transglucuronylation. The enzymes also catalyze self-transglycosylation to afford *O*-2- or *O*-3-linked disaccharide. The bovine enzyme showed the highest reactivity for providing a practical enzymatic approach to highly functional saccharides like compounds **184** and **185** bearing both carboxyl and sulfate groups in one molecule. The main drawback of this approach is the obtention of mixtures of 2-/3-linked disaccharides, however in the case of the sulfated galactopyranoside derivative the reaction proceeded with complete regioselectivity. 

## 3. Mannuronidation

**Scheme 46:**
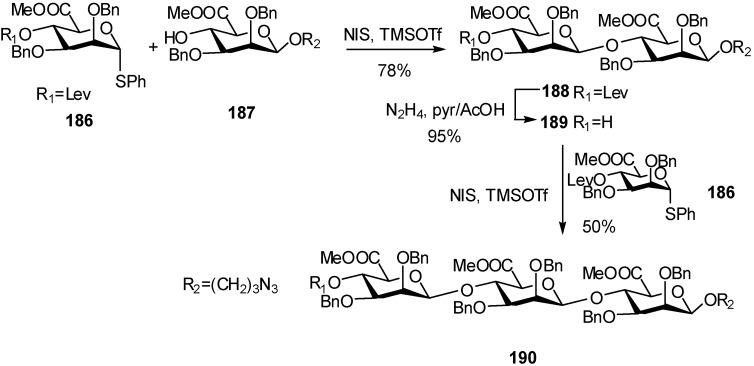
Preparation of a trisaccharide via mannuronidation.

Stereocontrolled synthesis of homooligomers of mannuronic acid was performed from thiomannuronic derivatives. In this alginate oligomer, the uronic acid monomers are interconnected through 1,4-interglycosidic linkages that have a 1,2-*cis* configuration. Preactivation of thiomannuronic donor **186** with NIS/TMSOTf followed by addition of the mannuronic acceptor gave the β-disaccharide **188** in 78% yield, which was subjected to the same coupling reaction with donor **186** [63]. The trisaccharide **190** was obtained in 50% yield (Scheme 46).

**Scheme 47:**
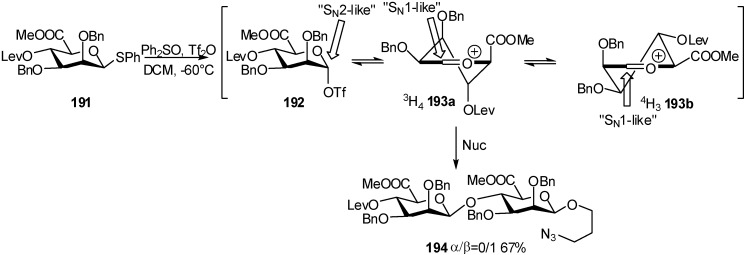
Proposed mechanism for mannuronidation from thiomannuronic donors.

Conformational studies on the thiomannuronic donors showed that the electron-withdrawing C-5 carboxylate group destabilized the oxocarbenium ion. The oxocarbenium intermediate would adopt a ^3^H_4_ half-chair conformation **193a**, with the carboxylate group in an axial position. The nucleophilic attack of the acceptor led to 1,2-*cis*-mannuronate **194** [64,65,66] (Scheme 47).

**Scheme 48:**
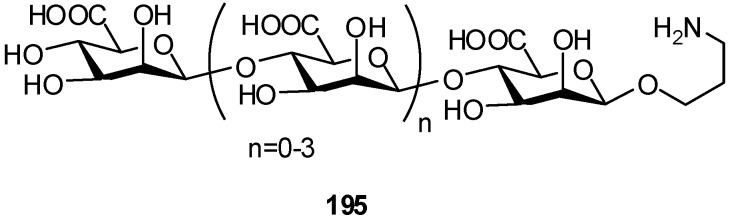
Oligomers with 1,2-*cis* linkage from mannuronate esters.

The stereodirecting effect of the C-5 glycuronate ester has been demonstrated in the synthesis of a set of oligomers of mannuronic acid, **195**, with 1,2-*cis* linkages (Scheme 48).

Further studies on conformationally restricted mannuronates **196** and **198** were enterprised to explore the stereoselectivity of 1,2-*cis-*glycosylation [67,68]. The stereoselectivity is dependent on the nature of the protecting groups on the mannose core.

**Scheme 49:**
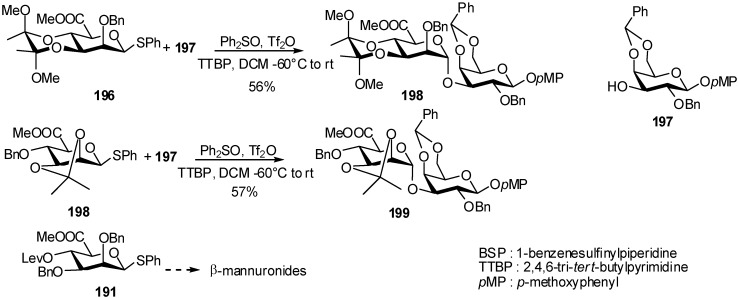
Constrained uronate donorsin mannuronidation reaction.

The flexible mannuronnate **191** gave excellent β-selectivity, whereas conformationnaly constrained uronate donors such as **196** and **198** provided predominantly α-linkage formation in pseudo-disaccharides **198** and **199** (Scheme 49).

## 4. Galacturonidation

**Scheme 50:**
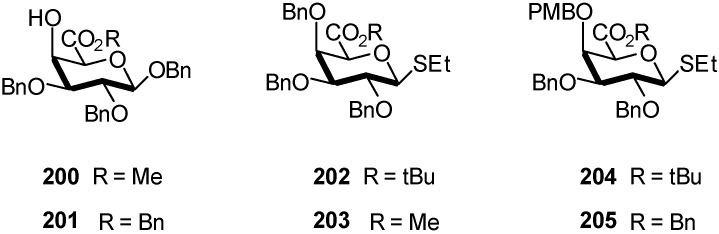
D-galacturonic acid ester derivatives as donors and acceptors.

Magaud *et al*. described for the first time, the stereoselective α-(1→4) glycosylation between two D-galacturonic acid ester derivatives (Scheme 50) giving rise to disaccharides in good yields [69].

Using *N*-iodosuccinimide-trifluoromethanesulfonic acid as promoter, the thioglycoside donor **203** reacted smoothly at low temperature (−60 °C), with the glycosyl acceptor **201** to give stereoselectively the α-(1→4) linked product **206** (Scheme 50) in very good yield (Table 1). The coupling between donor **202** and acceptor **200** was achieved under the same conditions, and furnished exclusively the dimer **207.**

**Table 1:** Results of galacturonidation of thioglycoside donors **202-205**.

Entry	Donor	Acceptor	Product	α/β ratio	Yield (%)
1	**203**	**201**	**206**	95/5	91
2	**202**	**200**	**207**	> 95/5	70
3	**204**	**201**	**208**	> 95/5	70
4	**205**	**201**	**209**	> 95/5	78

**Scheme 51:**
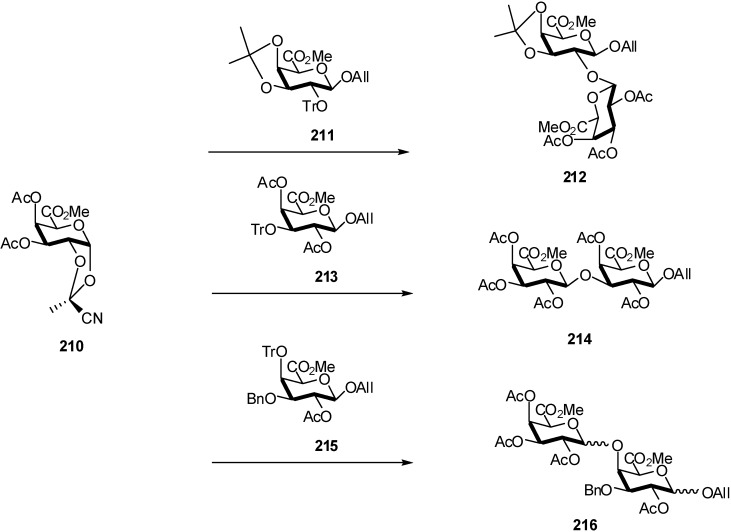
Stereoselective synthesis of β-linked disaccharides from cyano-orthoester donor.

Methyl 3,4-di-*O*-acetyl-l,2-*O*-[1-(exo-cyano)ethylidene]-α-D-galactopyranuronate was used as galacturonate donor by Vogel *et al*. [70]. The synthesis of disaccharides was carried out in the presence of tritylium perchlorate in dichloromethane. The coupling of **210** with **211** and **213** gave the expected β(1→2) and β(1→3)-linked disaccharides **212** (58%) and **214** (57%) containing no more than 1% of the α-anomer (Scheme 51). 

In the case of glycosylation at C-4 (reaction between of **210** and **215**) two phenomena were observed. First, l',2'*-trans* and l',2'*-cis*-disaccharides were formed, with the loss of stereoselectivity. Secondly, partial anomerisation at the glycosidic centre of the 1,2*-trans-*allyl glycoside **215** ocurred.

**Scheme 52:**
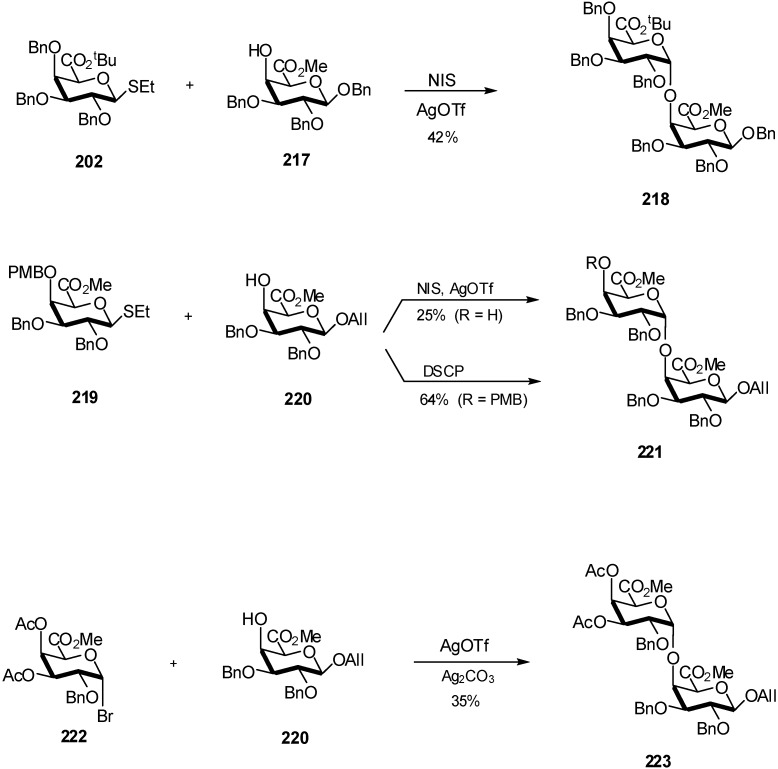
Preparation of homogalacturonanes.

Homogalacturonanes are interesting targets as their are the main components of plant pectin. Thioglycoside galacturonate donor **202** (Scheme 52) was tested with acceptor **217** using *N*-iodo-succinimide/silver triflate promotion at −20 °C with rigorous exclusion of moisture. The corresponding α-disaccharide **218** was obtained in 42% yield based on **217**, showing that the *tert*-butyl ester was not stable under the glycosylation reaction conditions.

During the glycosylation conditions described above using *p*-methoxybenzyl derivative **219** as glycosyl donor and compound **220** as acceptor, the *p*-methoxybenzyl protective group proved not to be stable and the corresponding disaccharide with a free hydroxyl group in the C-4'-position was isolated in only 25% yield. In order to manage the lability of the *p*-methoxybenzyl function, different promotors were checked. The best result was obtained with freshly prepared iodonium di-sym-collidine perchlorate (64% isolated yield based on **220**). Silver triflate/silver carbonate promoted glycosylation of glycosyl acceptor **220** with methyl(galactopyranosyluronate)bromide **222** provided the disaccharide **223** in 35% yield based on **220** [71]. This work by Vogel *et al*. allowed the preparation of di- and tri- galacturonan fragments. 

**Scheme 53:**
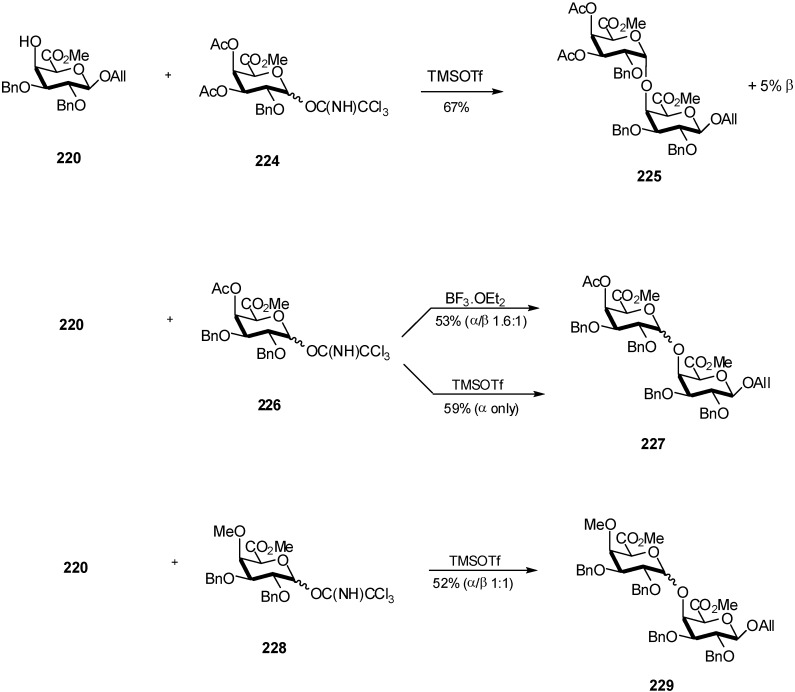
Preparation of oligogalacturonans.

On the other hand, the glycosylation of the galacturonate acceptor **220** with trichloroacetimidate donors in a ratio of 1:1 promoted by trimethylsilyl trifluoromethanesulfonate revealed a peculiar effect of the chosen substitution pattern (Scheme 53) [72]. Thus, the 3,4-di-*O-*acetyl-α-D-galacturonate trichloroacetimidate **224** provided 67% yield of the α-(1→4)-coupled disaccharide **225** and only 5% of the β-coupled disaccharide was detected. By way of contrast, the more active 2,3-di-*O*-benzyl glycosyl donor **226** coupled with **220** furnished the corresponding disaccharides **227** in a yield of 59% but no β-coupled disaccharide was observed. In earlier experiments the coupling of **220** with **226** in the presence of boron trifluoride diethyl etherate furnished the corresponding disaccharides **227** in a total yield of 53% and a disappointing α/β ratio of 1.6:1.

Finally, the methyl group in the *O*-4 position of the glycosyl donor **228** gave rise to the lowest stereoselectivity (nearly 1:1 for disaccharide **229**) with a total yield of 52%. Subsequent experiments shown that the α- or β-configuration of the trichloroacetimidate group at the anomeric center of the donors exerted no influence on the outcome of stereoselectivity of the glycosylations investigated. This approach using galacturonates suitable as donors in α-glycosylation reactions can be carried out directly from commercially available D-galacturonic acid avoiding the crucial oxidation step in comparison to an approach involving D-galactose-derived intermediates.

**Scheme 54:**
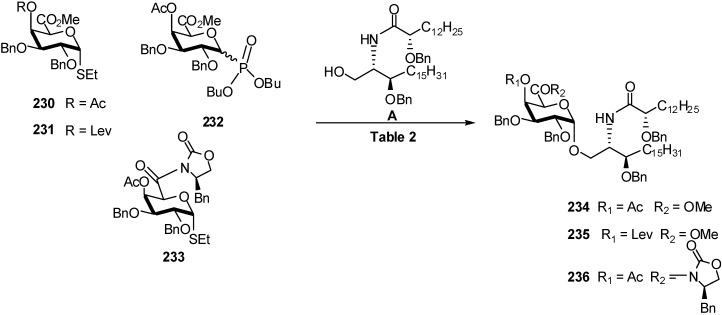
Synthesis of a conjugate of ceramide **A**.

The synthesis of glycosphingolipids is an important challenge. The Seeberger group studied the glycosylation of galacturonic acids **230** and **231** with ceramide **A**, to yield conjugate **234-236** (Table 2) [73] (Scheme 54). The problem in these reactions is the relatively poor solubility of ceramide **A** in many solvent systems at low temperature. During these studies, the well-known benefits of ether and the remote anchimeric assistance of C4 esters in galacto-configured systems in obtaining good α-selectivities became again apparent, as omission led to a dramatic increase in *β*-glycoside formation. A compromise between yield and selectivity was found by employing acetyl-protected thioglycoside **228** and the NIS/TfOH activator system. Thus, the product **234** was isolated in 85% yield and 4.2:1 selectivity with dioxane/toluene (3:1) as solvent. Both anomers were separated by flash column chromatography. 

**Table 2:** Effect of the activator system on galacturonidation of ceramide **A**.

Entry	Donor	Conditions	Product	α/β	Yield (%)
1	**233**	NIS/TBSOTf	**236**		< 10
2	**233**	NIS/TfOH	**236**	1.0:1	45
3	**231**	NIS/TfOH	**235**	2.0:1	49
4	**231**	DMTST	**235**		< 10
5	**232**	TMSOTf	**234**	2.1:1	96
6	**230**	NIS/TfOH	**234**	3.7:1	85
7	**230**	NIS/TfOH	**234**	4.2:1	85

Fischer glycosylation of free galacturonic acid was recently studied by Allam *et al*. Tests were first conducted using sulfuric acid as the catalyst [74]. The best yields (80%) were obtained using 10 equiv of octanol at 80 °C during 48 h with a catalytic amount of sulfuric acid. A lower excess of octanol (5 equiv) clearly decreased the overall yield.

The formation of furanosiduronate compounds has already been reported when galacturonic acid was treated with methanol in the presence of Amberlite IR-120H for 48 h at 35 °C in an orbital shaker affording methyl (methyl D-galactofuranosid)uronate as the only product, in a 2.6:1 β:α ratio [75].

**Scheme 55:**
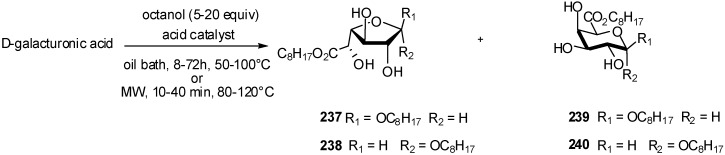
Fischer glycosylation of free galacturonic acid.

Shorter reaction times also resulted in lower yields. A higher temperature than 80 °C led to more important formation of degradation products (dark brown solution) while reaction at 50 °C showed a yield decrease. Several strong organic or mineral acid catalysts were used without noticeable changes in anomer ratio. In each case, the four anomers **237-238, 239-240** were present whatever the reaction conditions and the major one was identified as the β-furanose anomer (Scheme 55). The use of *p*-toluenesulfonic acid (PTSA) as the acid catalyst gave *n*-octyl (*n*-octyl D-galactoside) uronates **237-240** in good yield (83%), suggesting that organic sulfonic acids possess the appropriate acidities to promote the ester condensation. Under these experimental conditions, the ratio of the four anomers was approximately 50% of β-furanose **237**, 15% of α-furanose **238**, 5% of β-pyranose **239** and 30% of α-yranose **240** as determined by ^1^H NMR. After chromatography, the pure major β-furanose isomer **237** can be obtained in about 35% yield.

A significant acceleration of the reaction was observed using microwave activation, since all reactions were complete within 10 min. With H_2_SO_4_ as the catalyst, the best results were obtained with a 10-fold excess of octanol (20 equiv) at 100 °C. A lower temperature (80 °C) was detrimental since the yield in *n*-octyl (*n*-octyl D-galactosid)uronates **237-240** dramatically decreased (25%). On the contrary, with a higher temperature (120 °C), the reaction turned brown with partial degradation of the sugar compounds and lower yield (52%). When compared to thermal activation, yields and product purity were improved since the reaction medium was only slightly yellow after the reaction. Unfortunately, the ratio of the four anomers was only slightly modified by microwave activation: β-furanose **237** 60%, α-furanose **238** 15%, β-pyranose **239** 5%, and α-pyranose **240** 20%. In the presence of a catalytic amount of PTSA, *n*-octyl (*n*-octyl D-galactosid)uronates **237-240** were only obtained as the furanose isomers in 63% yield, the pyranose ones representing about 5% of the mixture. Several attempts to improve the reaction yield and to reduce the ratio of the α-furanose anomer were unsuccessful. A rapid esterification of the carboxylic group with PTSA, which proceeds faster than glycosylation, could favor the formation of the furanose ring.

## 5. Conclusions

Uronic acid derivatives are poor donors, due to the deactivating effect of the electron-withdrawing carboxylate group. Peracetylated bromides are easy to prepare and have been widely used as donors, however they are less reactive than imidates and can lead to the formation of by-products as glycals or orthoesters. In addition, trichloroacetimidates allow glycosylation conditions compatible with a large variety of protecting groups. Thioglycosides, iodides and phosphates have been also employed as donors, but they are less developed. Benzoyl protected donors prevents orthoester formation, whereas isobutyryl derivatives reduce the risk of transacylation to the acceptor, often observed when using acetyl protected donors. The use of 6,1-lactones is an alternative approach to GlcA glycosides, either in “classical” conditions or by MW-assisted procedures. Glucuronidation of aminosugars is especially difficult. NHAc-containing derivatives fail in these reactions, the most useful acceptors are azido precursors or NHTCA derivatives. Phenolic hydroxyls required the development of specific methods. As observed in general glycosylation reactions, both reactivity and stereoselectivity is highly dependent on the electronic and steric character of protecting groups. Finally, mannuronic and galacturonic acid glycosidations are less developed, probably due to their relative lower abundance in natural products, however analogous methodologies have been applied leading to comparable results. 
